# Metabolite profiling of *Trichinella spiralis* adult worms and muscle larvae identifies their excretory and secretory products

**DOI:** 10.3389/fcimb.2023.1306567

**Published:** 2023-12-08

**Authors:** Naphatsamon Uthailak, Poom Adisakwattana, Peerut Chienwichai, Phornpimon Tipthara, Joel Tarning, Charin Thawornkuno, Tipparat Thiangtrongjit, Onrapak Reamtong

**Affiliations:** ^1^ Department of Social and Environmental Medicine, Faculty of Tropical Medicine, Mahidol University, Bangkok, Thailand; ^2^ Department of Helminthology, Faculty of Tropical Medicine, Mahidol University, Bangkok, Thailand; ^3^ Princess Srisavangavadhana College of Medicine, Chulabhorn Royal Academy, Bangkok, Thailand; ^4^ Mahidol Oxford Tropical Medicine Research Unit, Faculty of Tropical Medicine, Mahidol University, Bangkok, Thailand; ^5^ Centre for Tropical Medicine and Global Health, Nuffield Department of Clinical Medicine, University of Oxford, Oxford, United Kingdom; ^6^ Department of Molecular Tropical Medicine and Genetics, Faculty of Tropical Medicine, Mahidol University, Bangkok, Thailand

**Keywords:** metabolomics, *Trichinella spiralis*, biomarker, excretory-secretory products, trichinellosis

## Abstract

Human trichinellosis is a parasitic infection caused by roundworms belonging to the genus *Trichinella*, especially *Trichinella spiralis*. Early and accurate clinical diagnoses of trichinellosis are required for efficacious prognosis and treatment. Current drug therapies are limited by antiparasitic resistance, poor absorption, and an inability to kill the encapsulating muscle-stage larvae. Therefore, reliable biomarkers and drug targets for novel diagnostic approaches and anthelmintic drugs are required. In this study, metabolite profiles of *T. spiralis* adult worms and muscle larvae were obtained using mass spectrometry-based metabolomics. In addition, metabolite-based biomarkers of *T. spiralis* excretory–secretory products and their related metabolic pathways were characterized. The metabolic profiling identified major, related metabolic pathways involving adenosine monophosphate (AMP)-dependent synthetase/ligase and glycolysis/gluconeogenesis in *T. spiralis* adult worms and muscle larvae, respectively. These pathways are potential drug targets for the treatment of the intestinal and muscular phases of infection. The metabolome of larva excretory–secretory products was characterized, with amino acid permease and carbohydrate kinase being identified as key metabolic pathways. Among six metabolites, decanoyl-l-carnitine and 2,3-dinor-6-keto prostaglandin F1α-d9 were identified as potential metabolite-based biomarkers that might be related to the host inflammatory processes. In summary, this study compared the relationships between the metabolic profiles of two *T. spiralis* growth stages. Importantly, the main metabolites and metabolic pathways identified may aid the development of novel clinical diagnostics and therapeutics for human trichinellosis and other related helminthic infections.

## Introduction

Trichinellosis is a parasitic disease caused by the infection of parasitic roundworms in the genus of *Trichinella* spp. (Pozio, 2007). This nematode possesses a wide range of host species such as human, mammals, reptiles, and birds (Gottstein et al., 2009). According to the Centers for Disease Control and Prevention (CDC; November 15, 2019), the annual human trichinellosis is estimated 10,000 cases worldwide caused by the ingestion of raw or undercooked meat, in particular *Trichinella* larvae- containing domestic pigs (Murrell and Pozio, 2011). Besides public health issues, *Trichinella* spp. also cause the significant economic losses of livestock production and food safety (Gottstein et al., 2009). Recently, 13 species of *Trichinella* spp. have been identified, including *T. spiralis*, *T. murrelli*, *T. native*, *T. patagoniensis*, *T. britovi*, *T. nelsoni*, *T. papuae*, *T. pseudospiralis*, *T. zimbawensis*, T6, T8, T9, and *T. chanchalensis* (Zarlenga et al., 2020). Among them, *T. spiralis* is known as the predominant species responsible for causing trichinellosis in humans and animals worldwide (Muñoz-Carrillo et al., 2018). Compared to other complicated life cycle roundworms, *Trichinella* spp. have a direct life cycle, which all stages are occurred in an individual host. Thus, the epidemiology of trichinellosis mainly transmitted by the consumption of *Trichinella*-infected meat (Gottstein et al., 2009). Regarding the clinical symptoms, human trichinellosis has been classified into two phases, including the enteral (intestinal) phase and a parenteral (muscular) phase (Gottstein et al., 2009). After the ingestion of infected meat, the larvae-containing collagen capsule called nurse cell (NC) is dissolved in the stomach by gastric acid and pepsin digestion. The muscle larvae (L1) are then released and invaded into the columnar epithelium in the small intestine, representing the intestinal phase of infection. Afterwards, adult worms are developed and the mating occurs approximately at day 7 post-infection. After new born larvae (NBL) are released, they migrate to various organs through the circulatory system, especially the musculoskeletal cells, where they are gradually encysted and developed into the muscular phase of infection (Kniel, 2013; Murrell, 2016; Muñoz-Carrillo et al., 2018).

Recently, three main criteria are available for the diagnosis of human trichinellosis: (1) the occurrence of clinical signs and symptoms; (2) the epidemiological examination; (3) the laboratory diagnoses, including the detection of L1 in muscle biopsy specimens and the detection of antibody against *Trichinella* spp. (Gottstein et al., 2009). However, the early diagnosis of human trichinellosis is still challenging due to non-pathognomonic clinical signs and symptoms, leading to the underestimation of infected cases and medical misclassification (Melese et al., 2021). Additionally, the detection of larvae in biopsy specimens and *Trichinella*-specific antibody are not sensitive enough for the early diagnosis of trichinellosis as the occurring of many false-negative cases (Dupouy-Camet and Bruschi, 2007; Bruschi et al., 2019). Hence, reliable diagnostic approaches for the early infection of human trichinellosis are required (Muñoz-Carrillo et al., 2018). Among various methods, biomarkers are widely used in the diagnosis of pathological conditions. It is also applicable in many research fields such as drug discovery and the monitoring of specific medication (Gromova et al., 2020). Although some previous research have been conducted to identify biomarkers specific to *T. spiralis*, most of them were focused on the protein-based biomarkers (Wang et al., 2013; Wang et al., 2014; Thawornkuno et al., 2022). On the other hand, only one study worked on the identification of metabolite-based biomarker from infected mice sera has been reported (Chienwichai et al., 2023). During the infection, parasitic nematodes release many excretory-secretory (ES) products with the immunomodulatory activity in order to escape host immune system. Since ES products play important roles in the survival of parasitic nematodes (Harnett, 2014), they can be considered as potential biomarkers for the diagnosis of parasitic diseases (Whitman et al., 2021). For instance, 14 potential metabolites were identified as candidate biomarkers for the detection of onchocerciasis, the infectious disease caused by the parasitic worm *Onchocerca volvulus* (Denery et al., 2010). In addition, the 2-methyl pentanoyl carnitine (2-MPC) was identified as a urine biomarker for the infection of *Ascaris lumbricoides* (Lagatie et al., 2020).

Currently, benzimidazole derivatives, especially albendazole and mebendazole, are common anthelmintic drugs widely used for the treatment of trichinellosis and other parasitic diseases. Both drugs affect the microtubule systems of parasites, resulting in the inhibition of glucose uptake and transportation, followed by cell death (Chai et al., 2021). Although the following drugs have been used continually, some limitations have been reported, involving the issue of drug resistance and the poor absorption in the gastrointestinal tract, especially intestinal lumen due to their low solubility (Dayan, 2003; Chai et al., 2021). Parasitic infection with roundworms such as *Trichinella spiralis* cause human trichinellosis. Drugs derived from benzimidazole are unable to kill the encapsulating muscle-stage larvae of *T. spiralis* (Pozio et al., 2001). Moreover, the failures of mebendazole to eliminate adult-stage worms in the small intestine and first-stage larvae in muscle have been reported (Pozio et al., 2001). Development of alternative anthelmintic drugs to overcome these limitations remains in progress, especially stage-specific drugs for adult worms and muscle larvae, which represent the intestinal and muscular phases of infection, respectively (Gottstein et al., 2009). Recently, metabolomic profiling has provided an understanding of the role of small molecules in various metabolic pathways at specific times and under certain conditions. Those condition-specific metabolites are related to critical biological processes of both parasites and hosts. Therefore, they represent potential targets in the development of novel anthelmintic drugs (Whitman et al., 2021). Although stage-specific antigens in *T. spiralis* adult worms have been reported (Yang et al., 2015), no studies have compared the stage-specific metabolic profiles of adult worms and muscle larvae.

Mass spectrometry-based metabolomics is a powerful method for the comprehensive characterization of metabolites in biological systems. It is applicable in many research areas, including biomarker discovery and drug development (Wishart, 2016; Alseekh et al., 2021). To date, few metabolite profiles of *T. spiralis* have become available (Chienwichai et al., 2023). Consequently, the stage-specific metabolomes of *T. spiralis* adult worms and muscle larvae were characterized in this study using mass spectrometry-based metabolomics and bioinformatics, which also identified metabolite-based biomarkers from their excretory–secretory (ES) products. This work provides fundamental metabolomic information which might be useful for the development of novel anthelmintic drugs and reliable diagnosis of trichinellosis.

## Materials and methods

### Ethical statement

Animal experiments were approved by the Faculty of Tropical Medicine Animal Care and Use Committee (FTM-ACUC), Mahidol University (FTM-ACUC 015/2021). Experiments were conducted in eight-week-old female ICR mice with all efforts to minimize the number of animals used for the reliable statistical analysis.

### Cultivation and maintenance of *T. spiralis*

By gastric gavage, 250 muscle larvae were used to infect each nine mouse models. To obtain adult worms, three mice were euthanized by carbon dioxide gas after being infected for seven days. By flushing the digestive tracts with normal saline, adult worms were collected and kept at -80°C (Ruenchit et al., 2022). For larvae preparation, the six infected mice were euthanized by carbon dioxide gas after 2 months of infection. L1 were released from the muscle tissue using pepsin digestion (0.7% HCl, 0.7% pepsin). Adult worms and larvae were washed by Phosphate-buffered saline (PBS) three times before metabolite extraction. The collection of ES products was adapted from previous work. Briefly, the larvae from three mice were cultured in RPMI medium for 24 hours at 37°C in order to collect ES (Wang et al., 2017).

### Metabolome extraction

The metabolome extraction was performed, according to the previous study (Chienwichai et al., 2023). For ES product, 20 µL of solution was mixed with 80 µL of cold methanol using vortex for 1 min. The mixture was incubated at 4°C for 20 min, following by centrifugation at 12,000 rpm for 10 min. Supernatant was collected and dried using a speed vacuum and stored at -80°C for further experiment. For *T. spiralis* adults and larvae, the worms were homogenized in 500 mL of methanol. After being frozen in liquid nitrogen and thawed, the tubes were centrifuged at 800 g for 1 min at 4°C. Supernatant was gathered and placed in a brand-new tube. This step was repeated to extract the pellet. The supernatant from the second extraction was mixed with the supernatant from the first extraction. The pellet was redissolved in 250 μL of deionized water, the pellet was frozen in liquid nitrogen and thawed. The supernatant was obtained by centrifugation at 15,000 g for 1 minute at 4°C and added to the earlier extraction. The collected supernatants were centrifuged at 15,000 g for one minute to remove any remaining debris. The clear supernatant was collected and dried in a speed vacuum (Tomy Digital Biology, Tokyo, Japan).

### Identification of metabolome using mass spectrometry

The metabolome was identified using mass spectrometry, according to the previous study (Chienwichai et al., 2023). Briefly, metabolome were identified using ultra high-performance liquid chromatography (UHPLC; Agilent 1260 Quaternary Pump, Agilent 1260 High Performance Autosampler and Agilent 1290 Thermostatted Column Compartment SL, Agilent Technologies, CA, USA) combined with a quadrupole time-of-flight mass spectrometer (Q-TOF MS; TripleTOF 5600+, SCIEX, US). The DuaSpray ion source was for the electrospray ionization (ESI). For UHPLC, 0.1% formic acid in water and 0.1% formic acid in acetonitrile were used as mobile phase A and B, respectively. Both mobile phases were mixed at the ratio 50:50 (v/v) and used for the suspension of samples. The mixture was transferred to a liquid chromatography vial and put in the auto-sampler. Samples (5 µL) was injected into the C18 reversed phase UHPLC column (ACQUITY UPLC BEH, 2.1 x 100 mm, 1.7 µM, Waters) with the flow rate of 0.3 mL/min at 40°C. For the UHPLC-Q-TOF MS system, Analyst Software version 1.7 (SCIEX) was used for the acquisition of mass ion chromatogram and mass spectra. The Q-TOF MS was operated in both positive and negative electrospray ionization modes (+ESI and -ESI). The information-dependent acquisition mode was performed with TOF-MS scan. Ten dependent product ion scans were performed in high sensitivity mode with the subtraction of dynamic background. Mass range of TOF-MS scan and product ion scan were 100-1000 and 50-1000 m/z, respectively. Each metabolite was equally aliquoted and pooled as a quality control (QC) sample. The system performance was evaluated by the injection of QC samples before, during and after sample analysis.

### Metabolomics analysis

Metabolomic-mass spectra files from the UHPLC-MS/MS (.wiff and.wiff.scan files) were processed using the XCMS online software version 3.7.1 (The Scripps Research Institute, CA, USA). Data from +ESI and -ESI modes were separately processed. Two different groups of each mode were compared using “Pairwise” mode with “UPLC/Triple TOF pos” protocol. Data from the XCMS were analyzed using MetaboAnalyst online software version 5.0 (https://www.metaboanalyst.ca/; Pang et al., 2021) in “Statistical Analysis (one factor)” module. The concentration of metabolites was filtered by “Interquantile range (IQR)”, follows by the normalization using quantile normalization, cube root data transformation, and data range scaling. The data visualization was performed using Principal Component Analysis (PCA), Partial Least Squares-Discriminant Analysis (PLS-DA), and Volcano plot. PCA and PL-DA were demonstrated with 95% confidence regions. Volcano plot was generated using log2 of fold change and -log of p-value. Differential metabolites were identified with the specific cut-off (fold-change>1.5 and p-value<0.01). The annotation of all potential metabolites was performed using XCMS online software.

### Pathway analysis

Pathway of interested metabolites was identified using the MetaboAnalyst online software version 5.0 (https://www.metaboanalyst.ca/; Pang et al., 2021) and the STITCH database version 5.0 (http://stitch.embl.de/; Szklarczyk et al., 2016). For MetaboAnalyst, the “Pathway analysis” module was selected with specific pathway analysis parameters. The hypergeometric test and relative-between centrality were chosen as the enrichment method and topology analysis, respectively. The pathway library of nematodes (*Caenorhabditis elegans*) was used for the analysis. For STITCH database, *T. spiralis* was selected as the organism of origin. All pathway analysis was identified with the p-value less than 0.01 as a statistical significance. The comparison of pathways in human and *T. spiralis* was performed using the KEGG PATHWAY Database (https://www.genome.jp/kegg/pathway.html).

### Identification of biomarkers

Potential biomarker candidates were identified by the MetaboAnalyst online software using the “Biomarker analysis” module. The concentration of metabolites was filtered by “Interquantile range (IQR)” and normalized using quantile normalization, cube root data transformation, and data range scaling. Data were then visualized using the receiver operating characteristic (ROC) with a statistical significance (fold-change>1.5 and p-value<0.01). The annotation of all potential metabolites-based biomarkers was performed using XCMS online software.

## Results

### Metabolite profiles of *T. spiralis* adult worms and muscle larvae

Metabolomes of *T. spiralis* at two different growth stages (adult worms and muscle larvae) were characterized using a quadrupole time-of-flight mass spectrometer in both positive and negative electrospray ionization modes. Based on XCMS software analysis, the 10485 features were identified in positive (5034 features) and negative (5451 features) ionization modes. The 20 most abundant metabolites in each growth stage of *T. spiralis* were identified and ranked according to the intensity of the corresponding features in the mass spectra (**Table 1**), and metabolic differences between adult worms and muscle larvae were found. Two of these metabolites, docosanamide and *N*-(2′-(4-benzenesulfonamide)-ethyl) arachidonoyl amine, were identified in both stages. Importantly, docosanamide was the most abundant metabolite in both stages. Thus, it might be an alternative biomarker for diagnosis of *T. spiralis* infection during the enteral and parenteral phases of worm development.

**Table 1:** Top 20 abundant identified metabolites from *T. spiralis* adult worms and muscle larvae with the highest intensity of mass spectra.

No.	Name	*m/z*	Retention time (min)	Mass error (ppm)	Adducted form	Mode
Top 20 abundant identified metabolites in adult worms						
1	Docosanamide	338.3437	14.54	3	M-H+	Positive
2	Ala Ile Gln Arg	504.3255	9.57	0	M+NH4	Positive
3	N-(2’-(4-benzenesulfonamide)-ethyl) arachidonoyl amine	504.3256	7.9	0	M+NH4	Positive
4	Ala Ile Ile Ile	393.2872	1.15	0	M+H-2H2O	Positive
5	5-HETrE	303.2325	11.12	0	M-H2O-H	Negative
6	Lys Lys Pro Val	435.3091	1.07	0	M+H-2H2O	Positive
7	13E-Docosenamide	336.3277	13.2	2	M-H+	Positive
8	Cys Phe Lys Gln	489.229	9.55	0	M+H-2H2O	Positive
9	CDP-N-methylethanolamine	425.0632	1.14	0	M+H-2H2O	Positive
10	1α,22-dihydroxy-23,24,25,26,27-pentanorvitamin D3	327.2322	10.76	0	M-H2O-H	Negative
11	Lys Lys Arg Thr	532.3566	7.81	0	M+H	Positive
12	Oleic Acid	281.2483	12.69	1	M-H	Negative
13	His Lys Arg	420.249	1.13	4	M-H2O-H	Negative
14	Nonadecyl oleate	538.5574	19.96	3	M+NH4	Positive
15	Glycerol 2-(9Z,12Z-octadecadienoate) 1-hexadecanoate 3-O-[alpha-D-galactopyranosyl-(1->6)-beta-D-galactopyranoside]	934.6445	14.59	2	M+NH4	Positive
16	Ala Ile Gln Arg	504.3252	10.76	0	M+NH4	Positive
17	Ala Ile Gln Arg	487.2989	7.82	0	M+H	Positive
18	2-Hexyldecanoic acid	255.2328	12.52	1	M-H	Negative
19	Ala Ile Gln Arg	487.2987	9.58	0	M+H	Positive
20	Mycocerosic acid (C29)	437.437	15.97	1	M-H+	Positive
Top 20 abundant identified metabolites in muscle larvae						
1	Docosanamide	338.3437	14.54	3	M-H+	Positive
2	PC(16:1(9Z)/22:2(13Z,16Z))	810.6043	0.16	3	M-H+	Positive
3	Ala Ile Gln Arg	504.3255	9.57	0	M+NH4	Positive
4	N-(2’-(4-benzenesulfonamide)-ethyl) arachidonoyl amine	504.3256	7.9	0	M+NH4	Positive
5	Ala Ile Ile Ile	393.2874	7.6	1	M+H-2H2O	Positive
6	PC(18:0/20:3(5Z,8Z,11Z))	810.6045	19.83	3	M-H+	Positive
7	Ala Phe Trp	387.1828	7.31	0	M+H-2H2O	Positive
8	D-Glucose 6-phosphate	259.0225	1.49	0	M-H	Negative
9	PC(18:0/20:4(5Z,8Z,11Z,14Z))	808.5884	18.8	3	M-H+	Positive
10	gamma-Glutamyl-beta-(isoxazolin-5-on-2-yl)alanine	365.1068	0.88	0	M+ACN+Na	Positive
11	DG(22:6(4Z,7Z,10Z,13Z,16Z,19Z)/22:6(4Z,7Z,10Z,13Z,16Z,19Z)/0:0)	754.5407	16.48	0	M+ACN+H	Positive
12	Lys Lys Pro Val	435.3091	1.07	0	M+H-2H2O	Positive
13	Ala Ile Ile Ile	393.2872	1.15	0	M+H-2H2O	Positive
14	PC(18:3(6Z,9Z,12Z)/20:2(11Z,14Z))	806.5724	17.55	2	M-H+	Positive
15	PS(20:1(11Z)/22:4(7Z,10Z,13Z,16Z))	830.5727	16.17	2	M+H-2H2O	Positive
16	PS(P-20:0/22:4(7Z,10Z,13Z,16Z))	834.6042	18.06	3	M+H-H2O	Positive
17	PC(16:0/22:5(4Z,7Z,10Z,13Z,16Z))	806.5731	16.72	3	M-H+	Positive
18	DG(22:5(4Z,7Z,10Z,13Z,16Z)/22:6(4Z,7Z,10Z,13Z,16Z,19Z)/0:0)	732.5571	18.86	1	M+NH4	Positive
19	PAF C16	522.3575	8.71	1	M-H+	Positive
20	PC(20:1(11Z)/22:6(4Z,7Z,10Z,13Z,16Z,19Z))	858.604	17.35	2	M-H+	Positive

**Figure 1:**
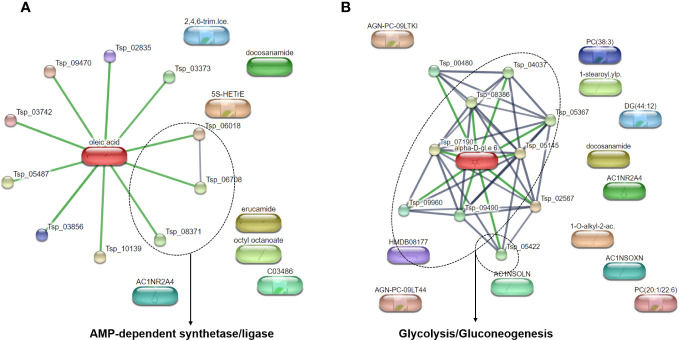
Pathway analysis of top 20 most abundant identified metabolites from *T. spiralis* **(A)** adult worms **(B)** muscle larvae analyzed by STITCH database.

To better understand the metabolic pathways related to the most abundant metabolites identified in *T. spiralis* adult worms and muscle larvae, these metabolites were included in a pathway analysis using the STITCH database (**Figure 1**). Based on statistical significance, pathways involving adenosine monophosphate (AMP)-dependent synthetase/ligase and glycolysis/gluconeogenesis were found to be related to the most abundant metabolites found in *T. spiralis* adult worms and muscle larvae, respectively. In the case of adult worms, AMP-dependent synthetase/ligase was identified as a related metabolic pathway, with a false discovery rate of 0.00101. In contrast, the glycolysis/gluconeogenesis pathway was related to the most abundant metabolites in muscle larvae, with a false discovery rate of 7.37×10^−13^. As expected, since adult worms and muscle larvae had only two of their most abundant metabolites in common, the metabolic pathways of both stages were different.

### Differential and biased metabolites in *T. spiralis* adult worms and muscle larvae

**Figure 2:**
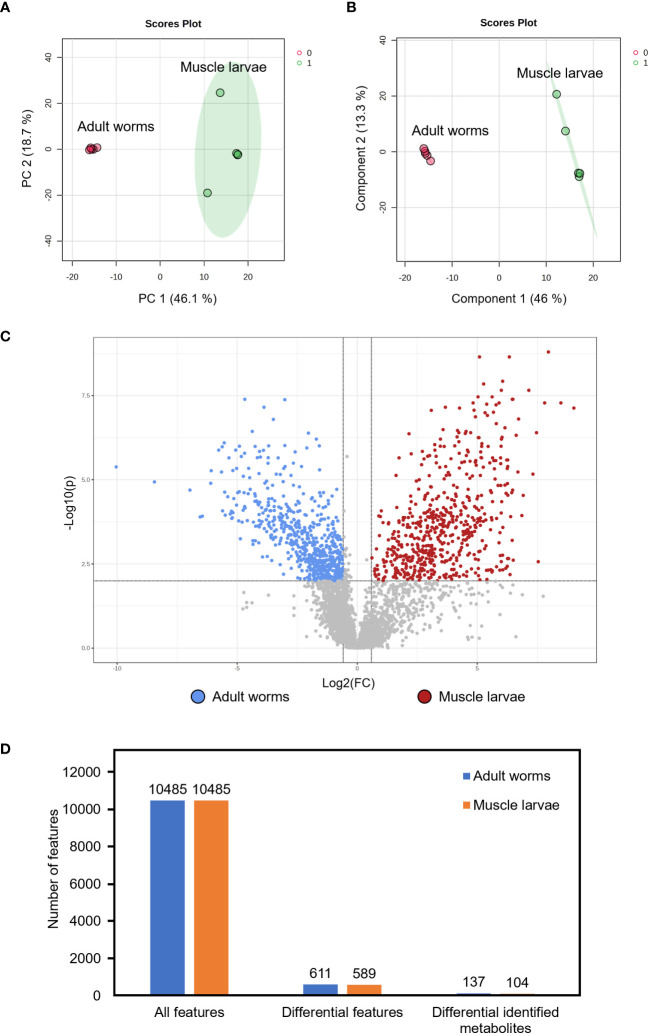
Identification of differential metabolites from *T. spiralis* adult worms and muscle larvae. **(A)** 2D scores plot of principal component analysis (PCA) and **(B)** Partial least squares discriminant analysis (PLS-DA) from the comparison between *T. spiralis* adult worms and muscle larvae. **(C)** Volcano plot analysis. Two vertical lines indicate a minimum of 1.5-fold changes. The horizontal line represents statistically significant at p-value <0.01. Dots above the horizontal line with the differences >1 and < -1 represent biased metabolites in muscle larvae and adult worms, respectively. **(D)** Number of features from metabolomics analysis. Data were analyzed with fold-change >1.5 and p-value <0.01).

To compare the metabolomes of *T. spiralis* adult worms and muscle larvae, metabolomics data obtained from mass spectrometry in positive and negative electrospray ionization modes were combined and principal component analysis (PCA) and partial least squares discriminant analysis (PLS-DA) was performed using the MetaboAnalyst online software (**Figures 2A, B**). Notably, two clusters were clearly separated, indicating the presence of stage-specific metabolites adult worms and muscle larvae. To identify these stage-biased metabolites, volcano plots were created and statistical significances (|fold change| >1.5, *p* < 0.01) were identified (**Figure 2C**). Among 10485 features in the mass spectra, statistically significant differences were identified among 1200 of these features, which could be divided into adult worm-biased metabolites (611 features) and muscle larva-biased metabolites (589 features). Among these, 137 and 104 features were identified as potential biased metabolites in adult and muscle larvae stages of *T. spiralis*, respectively (**Figures 2D**; **Supplementary Table S1**). The 20 most abundant biased metabolites from *T. spiralis* adult worms and muscle larvae are shown in **Table 2**.

**Table 2:** Top 20 biased metabolites identified from *T. spiralis* adult worms and muscle larvae with the highest fold change relative to different stages of *T. spiralis*.

No.	Potential metabolites	*m/z*	Mass error (ppm)	Adducted form	Mode	Fold change	P-value
Top 20 biased metabolites identified from *T. spiralis* adult worms							
1	DG(15:0/18:2(9Z,12Z)/0:0)	559.4701	4	M-H2O-H	Negative	348.58	1.162E-05
2	Ile Met Pro Arg	514.2828	2	M-H	Negative	125.38	2.024E-05
3	DG(17:2(9Z,12Z)/20:4(5Z,8Z,11Z,14Z)/0:0)	607.471	3	M-H2O-H	Negative	93.34	0.0001267
4	Ile Met Pro Arg	514.2828	2	M-H	Negative	54.51	1.315E-06
5	Cys Gln Arg Trp	572.2411	1	M-H2O-H	Negative	50.24	5.894E-06
6	Ile Met Pro Arg	514.282	1	M-H	Negative	49.27	1.046E-06
7	Cys Phe His Ile	560.2651	0	M+ACN+H	Positive	48.81	8.361E-05
8	DG(15:0/18:0/0:0)	563.5012	5	M-H2O-H	Negative	45.12	0.0001121
9	Ile Ile Ile Gln	466.305	4	M-H2O-H	Negative	41.99	3.193E-05
10	2-hydroxyphytanic acid	309.279	1	M-H2O-H	Negative	36.86	0.0001337
11	Ile Ile Met Tyr	538.2825	0	M+	Positive	30.11	2.258E-05
12	1α,22-dihydroxy-23,24,25,26,27-pentanorvitamin D3	327.2322	0	M-H2O-H	Negative	26.24	2.418E-05
13	Ile Met Pro Arg	514.2818	0	M-H	Negative	25.84	6.708E-05
14	Sphingosine	298.2755	1	M-H+	Positive	25.69	4.084E-08
15	2alpha,3alpha-(Difluoromethylene)-5alpha-androstan-17beta-ol acetate	347.2186	0	M-H2O-H	Negative	22.98	4.598E-06
16	9,14,19,19,19-pentadeuterio-1α,25-dihydroxyprevitamin D3	466.3311	0	M+2Na-H	Positive	20.71	3.661E-07
17	PC(O-18:0/O-1:0)	522.3933	1	M-H+	Positive	19.26	2.604E-05
18	Ile Ile Met Tyr	538.2826	0	M+	Positive	18.10	4.473E-05
19	14R-hydroxy-11E-eicosenoic acid	307.2632	1	M-H2O-H	Negative	17.50	0.0002358
20	Oleic Acid	281.2483	1	M-H	Negative	17.26	5.563E-05
Top 20 biased metabolites identified from *T. spiralis* muscle larvae							
1	PC(18:0/20:4(5Z,8Z,11Z,14Z))	808.5884	3	M-H+	Positive	105.31	1.60E-07
2	DG(22:6(4Z,7Z,10Z,13Z,16Z,19Z)/22:6(4Z,7Z,10Z,13Z,16Z,19Z)/0:0)	754.5407	0	M+ACN+H	Positive	89.45	4.10E-08
3	PS(20:1(11Z)/22:4(7Z,10Z,13Z,16Z))	830.5727	2	M+H-2H2O	Positive	87.82	4.10E-08
4	PC(20:1(11Z)/22:6(4Z,7Z,10Z,13Z,16Z,19Z))	858.604	2	M-H+	Positive	80.69	2.20E-09
5	PC(18:0/20:3(5Z,11Z,14Z))	810.6045	3	M-H+	Positive	77.12	2.60E-06
6	Benzo[a]pyrene-7,8-dihydrodiol-9,10-oxide	366.11	0	M+ACN+Na	Positive	77.06	4.10E-06
7	PS(P-20:0/22:4(7Z,10Z,13Z,16Z))	834.6042	3	M+H-H2O	Positive	75.91	4.60E-06
8	PC(16:0/22:5(4Z,7Z,10Z,13Z,16Z))	806.5731	3	M-H+	Positive	66.55	1.20E-08
9	PC(18:3(6Z,9Z,12Z)/20:2(11Z,14Z))	806.5724	2	M-H+	Positive	64.75	4.50E-05
10	Adenosine monophosphate	346.0556	0	M-H	Negative	61.57	0.000374
11	PI(O-20:0/16:0)	835.6077	1	M+H-H2O	Positive	46.91	2.11E-05
12	D-Glucose 6-phosphate	259.0225	0	M-H	Negative	46.90	0.000306
13	Spermidine	146.1652	0	M+H	Positive	42.33	0.009069
14	PS(P-20:0/22:6(4Z,7Z,10Z,13Z,16Z,19Z))	830.5711	1	M+H-H2O	Positive	40.06	1.02E-07
15	DG(22:6(4Z,7Z,10Z,13Z,16Z,19Z)/22:6(4Z,7Z,10Z,13Z,16Z,19Z)/0:0)	730.5405	0	M+NH4	Positive	38.46	1.42E-08
16	PS(O-18:0/22:6(4Z,7Z,10Z,13Z,16Z,19Z))	804.5555	1	M+H-H2O	Positive	35.35	1.48E-06
17	2-Protocatechoylphloroglucinolcarboxylate	348.0715	0	M+ACN+H	Positive	34.76	0.000166
18	DG(22:5(4Z,7Z,10Z,13Z,16Z)/22:6(4Z,7Z,10Z,13Z,16Z,19Z)/0:0)	732.5571	1	M+NH4	Positive	32.73	3.45E-08
19	PS(21:0/0:0)	566.3441	4	M-H	Negative	30.33	0.002262
20	PS(12:0/13:0)	636.3866	3	M-H	Negative	30.20	0.000003

**Figure 3:**
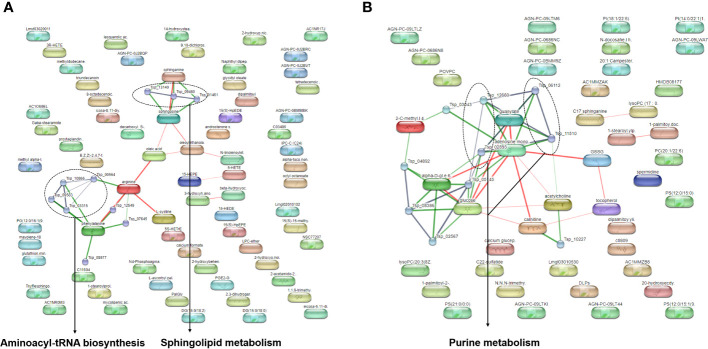
Pathway analysis of biased-metabolites identified from *T. spiralis* **(A)** adult worms **(B)** muscle larvae after analyzed by the STITCH database.

**Figure 4:**
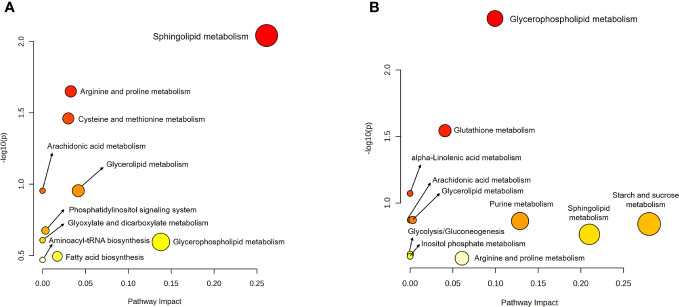
Pathway analysis of biased-metabolites identified from *T. spiralis* **(A)** adult worms **(B)** muscle-stage larva analyzed using the MetaboAnalyst software.

**Figure 5:**
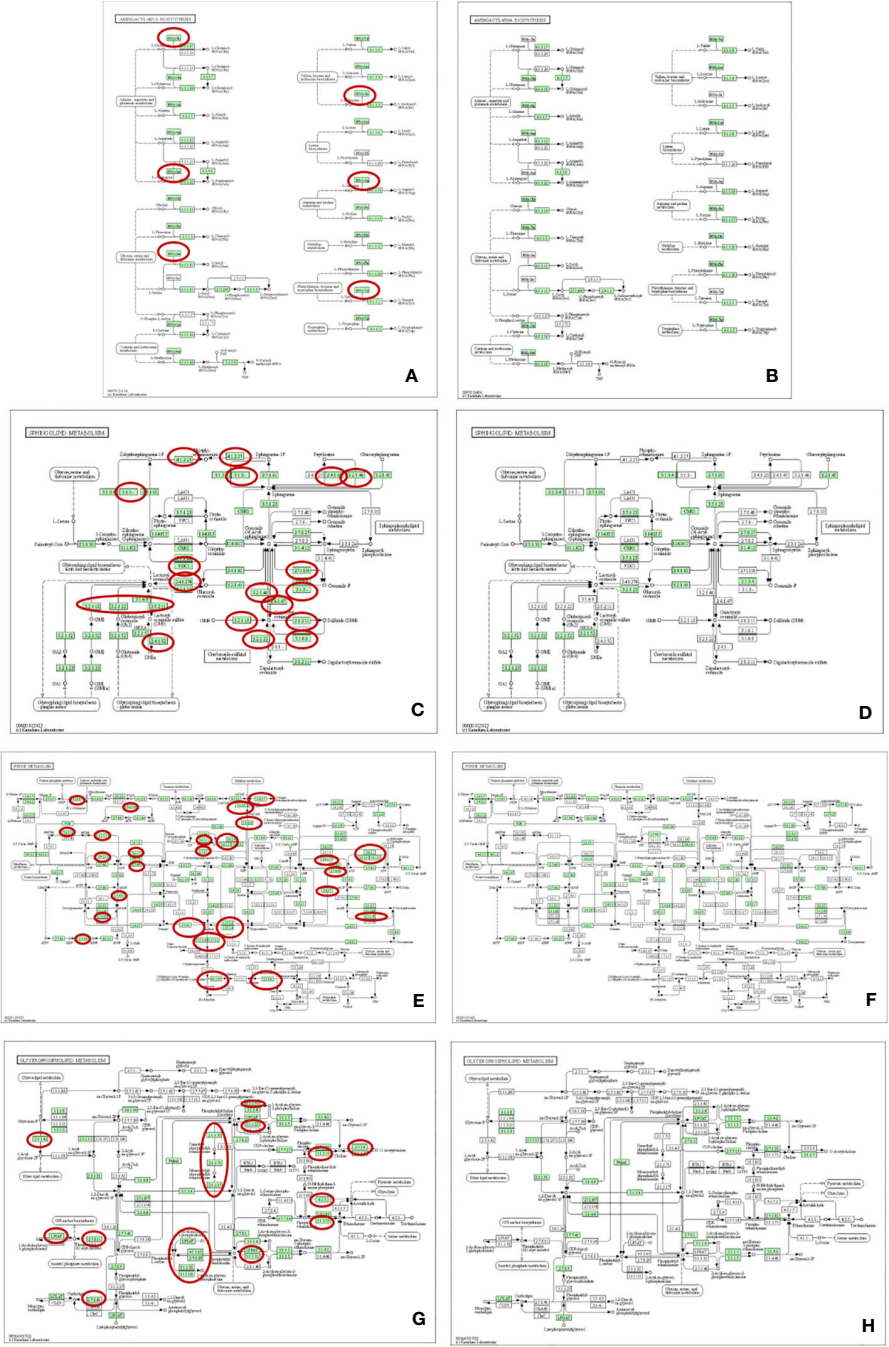
Pathway analysis using KEGG databases in human **(A, C, E, G)** and *T. spiralis* **(B, D, F, H)**. **(A, B)** Aminoacyl-tRNA biosynthesis; **(C, D)** Sphingolipid metabolism; **(E, F)** Purine metabolism; **(G, H)** Glycerophospholipid metabolism. Red circles indicating the biological molecules found only in human pathways.

For better understanding of related metabolic pathways of stage-biased metabolites in *T. spiralis*, pathway analysis was performed using the STITCH database (**Figure 3**) and MetaboAnalyst software (**Figure 4**). Based on STITCH analysis, metabolites of aminoacyl-tRNA biosynthesis and sphingolipids were enriched in adult worms, with false discovery rates of 0.00545 and 0.00554, respectively. In contrast, muscle larva-biased purine metabolites were identified with a false discovery rate of 0.027. MetaboAnalyst pathway analysis revealed that sphingolipid metabolism and glycerophospholipid metabolism were significant pathways related to the adult-biased and muscle larva-biased metabolites, respectively. Notably, both software analyses indicated that sphingolipid metabolism was the predominant pathway related to adult-biased metabolites. Using the Kyoto Encyclopedia of Genes and Genomes (KEGG) database, four pathways (aminoacyl-tRNA biosynthesis, sphingolipid metabolism, purine metabolism, and glycerophospholipid metabolism) were found to be common to humans and *T. spiralis*. However, the pathways in each species involved different numbers of biological molecules (**Figure 5**; **Supplementary Table S2**). Seven molecules were found to be involved only in human aminoacyl-tRNA biosynthesis (tRNA-Glu, tRNA-Asn, tRNA-Ser, mitochondrial methionyl-tRNA formyltransferase, tRNA-Ile, tRNA-Arg, tRNA-Tyr), and 12 were involved only in human sphingolipid metabolism (sphingosine-1-phosphate lyase 1, sphingosine-1-phosphate phosphatase 2, ceramide galactosyltransferase, galactosylceramidase, alkaline ceramidase 3, ceramide kinase, β-1,4-galactosyltransferase 6, neuraminidase 3, galactosidase alpha, arylsulfatase A, galactose-3-*O*-sulfotransferase 1, β-1,4-*N*-acetyl-galactosaminyltransferase 1). Additionally, we identified 19 biological molecules involved in human purine metabolism (phosphoribosyl pyrophosphate amidotransferase, Nudix hydrolase 16, phosphoribosylaminoimidazolecarboxamide formyltransferase, adenine phosphoribosyltransferase, prune exopolyphosphatase 1, guanosine-3′,5′-bis(diphosphate) 3′-pyrophosphohydrolase, apyrase, guanylate kinase 1, deoxycitidine/deoxyadenosine/deoxyguanosine kinase, guanosine monophosphate reductase, adenine phosphoribosyltransferase, guanine deaminase, xanthine dehydrogenase, 2-oxo-4-hydroxy-4-carboxy-5-ureidoimidazoline decarboxylase, allantoicase, adenosinetriphosphatase, nucleoside-triphosphatase, adenylate kinase 3, and deoxycytidine kinase) and 15 molecules involved in glycerophospholipid metabolism (glyceronephosphate *O*-acyltransferase, manganese-dependent ADP-ribose/CDP-alcohol diphosphatase, lecithin-cholesterol acyltransferase, phospholipase A and acyltransferase 3, phosphatidylethanolamine *N*-methyltransferase, choline *O*-acetyltransferase, phosphoethanolamine/phosphocholine phosphatase, ethanolamine-phosphate phospho-lyase, ethanolamine-phosphate cytidylyltransferase, lysophospholipid acyltransferase 5, phosphatidylserine decarboxylase, phosphatidylserine synthase 2, phospholipase A1 member A, CDP-diacylglycerol-inositol 3-phosphatidyltransferase, and cardiolipin synthase 1). Overall, the molecular pathways in humans are more complicated than those of *T. spiralis* because the former involves a larger number of biological molecules.

### Identification of biomarkers from ES products of *T. spiralis* larvae

**Figure 6:**
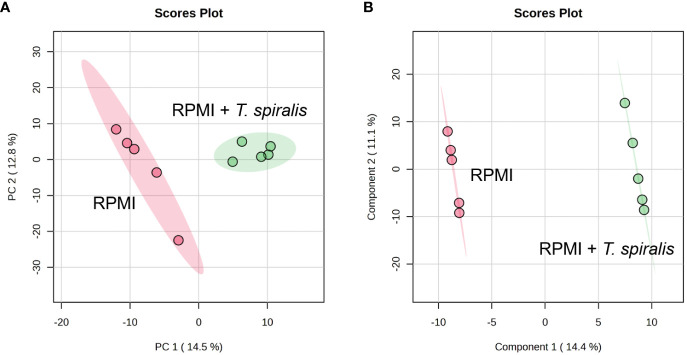
2D Scores plot of PCA **(A)** and PLS-DA **(B)** analysis of metabolome identified from RPMI media with and without *T. spiralis*.

**Figure 7:**
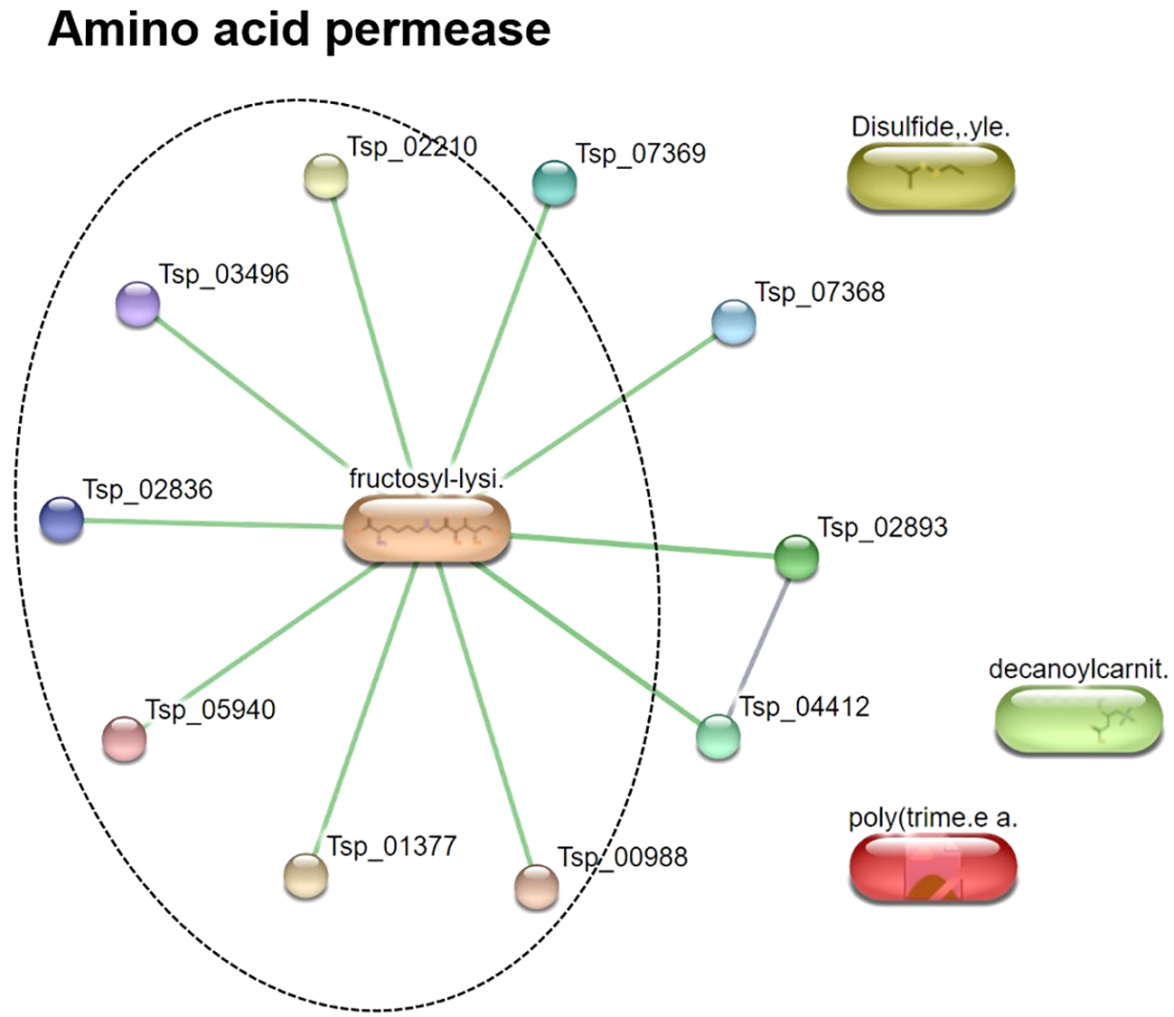
Pathway analysis of metabolite-based ES products released from *T. spiralis* after cultivation in RPMI media. Data were acquired using the STITCH database.

To characterize the *T. spiralis* ES substances, the metabolome of *T. spiralis* was extracted after cultivation in RPMI medium, and quadrupole time-of-flight mass spectra of the extract were compared with those of the RPMI medium alone. PCA and PLS-DA identified significant differences between the metabolomes extracted from muscle larvae and adult worms, resulting in the clear separation of two clusters (**Figure 6**). These results indicated that some metabolite-based ES products were released into the RPMI medium. Biomarker analysis using MetaboAnalyst was further conducted to establish more potential metabolite-based biomarker candidates from ES products of *T. spiralis*. Among the 19863 mass spectra obtained in RPMI medium with and without *T. spiralis*, features corresponding to six molecules were identified as potential biomarkers (fold change > 1.5 and *p* < 0.01) using classical univariate receiver operating characteristic curve analysis. These molecules included fructoselysine, dipropyl hexanedioate, decanoyl-l-carnitine, ethyl isopropyl disulfide, 2,3-dinor-6-keto-prostaglandin F1α-d9, and phenol-formaldehyde, cross-linked, triethylenetetramine activated (**Table 3**). To identify the related metabolic pathways, six candidate biomarkers found in ES products of *T. spiralis* larvae were analyzed using the STITCH database (**Figure 7**), and amino acid permease metabolism was identified as a potential pathway related to those candidate biomarkers, with a false discovery rate of 5.46×10^−13^.

**Table 3:** Biomarker analysis of ES products produced from *T. spiralis* after cultivation in RPMI media.

No	Potential metabolites	*m/z*	Retention time (min)	Mass error (ppm)	Adducted form	Mode	AUC	P-value	Fold change
1	Fructoselysine	307.1507	1.47	1	M-H	Negative	1.00	0.00184	1.62
2	Dipropyl hexanedioate	229.1446	13.3	0	M-H	Negative	1.00	0.00690	1.50
3	Decanoyl-L-carnitine	316.2483	16.28	0	M+H	Positive	1.00	0.00410	-1.91
4	Ethyl isopropyl disulphide	117.0198	0.95	1	M-H2O-H	Negative	1.00	0.00002	-7.61
5	2,3-dinor-6-keto Prostaglandin F1α-d9	316.2479	15.9	0	M+H-2H2O	Positive	0.88	0.00609	-1.65
6	Phenol-formaldehyde, cross-linked, triethylenetetramine activated	334.2213	9.26	0	M+ACN+Na	Positive	0.80	0.00606	-1.69

Data were analyzed with specific cut-off (fold-change>1.5 and p-value<0.01).

## Discussion

Trichinellosis is a parasitic disease that widely affects both public health and livestock-producing economies (Gottstein et al., 2009). Since early diagnosis and treatment of human trichinellosis remains problematic (Pozio et al., 2001; Dayan, 2003; Dupouy-Camet and Bruschi, 2007), reliable biomarkers and stage-specific targets are required to enable alternative diagnoses and development of potential anthelmintic drug treatments for trichinellosis.

In this study, the most abundant metabolites in *T. spiralis* adult worms and muscle larvae were identified using mass spectrometry-based metabolomics. As only two molecules were common to the 20 most abundant metabolites found in each growth stage, adult worms and muscle larvae are expected to have different biological activities corresponding to their different physical appearances. To support this hypothesis, metabolic pathway analysis of the 20 most abundant metabolites identified in two consecutive *T. spiralis* growth stages was performed using the STITCH database. As expected, AMP-dependent synthetase/ligase and glycolysis/gluconeogenesis were identified as major, related metabolic pathways with the lowest false discovery rate in adult worms and muscle larvae, respectively. AMP is an important cellular metabolite produced from various enzymatic reactions and regulates signal transduction and intracellular energy transfer (Mondal et al., 2017). Since adult worms require energy for the production of eggs and regulation of the neuromuscular system (Harder, 2016), AMP-dependent synthetase/ligase may be part of a major metabolic pathway linked to the most abundant metabolites in adult worms. AMP-dependent synthetase/ligase was also identified as a stage-specific protein in the early adult growth stage of *Haemonchus contortus*, a parasitic nematode in ruminants (Gadahi et al., 2016). In contrast, both glycolysis and gluconeogenesis are involved in glucose metabolism, which is relatively high during nurse cell formation in newborn larvae (Wu et al., 2013). Many enzymes involved in glycolysis and gluconeogenesis have previously been identified in third-stage larvae of *Caenorhabditis elegans* (O'Riordan and Burnell, 1989). Antimalarial drug that target the glycolytic enzymes of parasites, such as artesunate, quinine, and mefloquine, have demonstrated notable anti-leishmanial effectiveness recently (Yousef et al., 2020). Furthermore, suramin has been shown by Khanra et al. to be an effective and efficient anti-leishmanial medication (Khanra et al., 2020). It has been demonstrated that suramin blocks the glycolytic enzymes of *Trypanosoma brucei*, the parasite that causes African trypanosomiasis, or sleeping sickness (von der Ahe et al, 2018). Therefore, glycolysis/gluconeogenesis might be a fascinating target to treat *T. spiralis* larvae.

According to our previous study, (Chienwichai et al., 2023) the metabolomics analysis of *T. spiralis*-infected mouse sera has been reported. We found that D-glucose 6-phosphat found in adult worms and larvae was also detected in the *T. spiralis*-infected mouse sera at 4 weeks post-infection. In addition, two *T. spiralis* larvae metabolites, [PS(12:0/15:0) and DG(13:0/18:3(9Z,12Z,15Z)/0:0)], were also presented in *T. spiralis*-infected mice sera at both 2 and 8 weeks post-infection. These metabolites might be originated from the parasite and distributed to the host blood circulation. One of limitation in this research was indistinguishability between host and parasite metabolites. Although several washing steps by PBS were performed in this experiment when prepared the *T. spiralis* adult worms and larvae, there were possibility to obtain host metabolites in the sample. Therefore, the identification of the metabolite sources are unachievable by using this approach.

Similar to the predominant metabolites, the metabolomes in adult worms and muscle larvae were different, as evidence by the significant separation of two sample clusters identified using PCA and PLS-DA. Based on a volcano plot, all biased metabolites in *T. spiralis* adult worms and muscle larvae were identified and included in pathway analyses. Statistical analyses identified two predominant metabolic pathways related to adult-biased metabolites, namely those associated with sphingolipid metabolism and aminoacyl-tRNA biosynthesis. Sphingolipid metabolism is a vital cellular pathway involving the formation of various sphingolipids and ceramide, a central component in the pathway (Pralhada Rao et al., 2013). Additionally, sphingolipids are vital components of biological membranes in eukaryotic organisms and play roles in fundamental biological processes. The key roles of sphingolipid metabolism in facilitating development and aging processes in nematodes have been previously reported for *C. elegans* (Cutler et al., 2014). Since genetic and pharmacological inhibition of sphingolipid synthesis slow the rate of development and prolong the egg laying period in *C. elegans*, sphingolipid metabolism is an essential process in adult nematodes. In this study, sphingolipid metabolites were predominantly detected in *T. spiralis* adult worms and concluded to be part of a metabolic pathway that is key to their physical development, reproduction, and survival. Besides sphingolipid metabolism, evidence of aminoacyl-tRNA biosynthesis was also identified from adult-biased metabolites. In biological systems, aminoacyl-tRNAs are substrates involved in protein translation (Ibba and Söll, 2000). Proteins are one of the major structural components in nematodes, especially in the cuticles (Fetterer and Rhoads, 1993). Hence, adult worm-biased metabolites might belong to the aminoacyl-tRNA biosynthesis pathway, which is important during the physical development of *T. spiralis*. In the case of muscle larva-biased metabolites, two pathways involving purine metabolism and glycerophospholipid metabolism were identified. In general, nematodes are unable to synthesize purines; therefore, all purine precursors are acquired from their host (Harder, 2016). During the muscular phase of infection, newborn larvae form a capsule with a collagenous wall (a “nurse cell”) to protect the parasite and regulate their metabolism (Wu et al., 2008). Metabolism of protein, glucose, and lipid is faster during nurse cell formation than in other growth stages (Wu et al., 2013). Therefore, the relationship between muscle larva-biased metabolites and glycerophospholipid metabolism observed in this study was consistent with previous research that identified glycerophospholipids as a predominant lipid class in muscle larvae of *Trichinella papuae* (Mangmee et al., 2020). KEGG database analysis identified many biological molecules in human but not *T. spiralis* pathways. This may be a consequence of the complexity of humans because nematodes lack many human biological systems, such as discrete circulatory and respiratory systems (Basyoni and Rizk, 2016). For an application, parasitic metabolites are vital to the survival of parasites since they contain important enzymes and substrates from a variety of metabolic pathways. Therefore, the stage-specific metabolites might be potential targets in the development of novel anthelmintic drugs as inhibitors of essential metabolic pathways (Taylor et al., 2013; Whitman et al., 2021).

The ES products released by parasitic nematodes play an important immunomodulatory role. They assist the survival and evasion of parasites from host immune system, resulting in long-term infection (Harnett, 2014). In this study, we attempted to identify ES product-based biomarker candidates that might enable alternative diagnoses of *T. spiralis* infection. Among 19863 mass spectra, six features were potential metabolite-based biomarkers, particularly decanoyl-l-carnitine, and 2,3-dinor-6-keto prostaglandin F1alpha-d9. l-Carnitine is fundamental in lipid metabolism, with different roles in cell maintenance, especially fatty acid transfer between organelles (Jacques et al., 2018). It is secreted from helminths during their life cycle and has anti-inflammatory and antioxidant properties (Keskin et al., 2015; Lee et al., 2015; Yeshi et al., 2022). In contrast to l-carnitine, prostaglandins play essential roles in inflammatory responses leading to tissue inflammation (Ricciotti and FitzGerald, 2011). Prostaglandins are released from cercariae of *Schistosoma mansoni* and play a major role in the release of histamine from the host in response to cercarial penetration (Salafsky et al., 1984; Yeshi et al., 2022). Additionally, prostaglandins found in *S. mansoni* eggs assist the migration of cercariae inside human hosts (Ramaswamy et al., 2000; Giera et al., 2018; Yeshi et al., 2022). Since prostaglandins are important for initiating penetration of cercariae, 2,3-dinor-6-keto prostaglandin F1α-d9 might be an alternative biomarker for the diagnosis of trichinellosis. Based on pathway analysis of six candidate biomarkers, amino acid permease was identified as a relevant metabolic pathway. Amino acid permeases are amino acid transporters found in fungi, plants, and animals (Wipf et al., 2002) and might be related to the high protein metabolism that occurs during nurse cell formation in newborn larvae (Wu et al., 2013). We attempted to check the six potential metabolites from this study by comparing to the metabolomes of *Caenorhabditis elegans* reported in the previous study (Molenaars et al., 2021). The results show that all 6 potential metabolites have not been reported in the *C. elegans*. Therefore, they might be considered as potential biomarkers. Although six metabolites were identified among *T. spiralis* ES products and may be potential biomarkers for trichinellosis, further studies should be performed to evaluate their specificity for future clinical diagnostics development.

In conclusion, comprehensive metabolic profiles of stage-specific *T. spiralis*, specifically adult worms and muscle larvae, were obtained in this study. The related metabolic pathways of the stage-biased metabolites were also predicted using bioinformatics. Additionally, statistical analyses identified six biomarker candidates among the ES products released from *T. spiralis* larvae. These findings may aid the development of novel clinical diagnostics and anthelmintic therapies for trichinellosis.

## Data availability statement

The data presented in the study are deposited in the Science Data Bank repository, accession number Ñ0.57760/sciencedb.11891 (https://www.scidb.cn/en/anonymous/bmFhdVFq).

## Ethics statement

The animal study was approved by Faculty of Tropical Medicine Animal Care and Use Committee (FTM-ACUC 015/2021), Mahidol University. The study was conducted in accordance with the local legislation and institutional requirements.

## Author contributions

NU: Conceptualization, Data curation, Formal analysis, Investigation, Methodology, Validation, Visualization, Writing – original draft, Writing – review & editing. PA: Conceptualization, Methodology, Resources, Writing – review & editing. PC: Data curation, Methodology, Writing – review & editing. PT: Data curation, Methodology, Writing – review & editing. JT: Conceptualization, Resources, Writing – review & editing. CT: Methodology, Writing – review & editing. TT: Methodology, Writing – review & editing. OR: Conceptualization, Data curation, Formal analysis, Funding acquisition, Investigation, Visualization, Writing – original draft, Writing – review & editing.
